# Recurrent duplications of the annexin A1 gene (*ANXA1*) in autism spectrum disorders

**DOI:** 10.1186/2040-2392-5-28

**Published:** 2014-04-10

**Authors:** Catarina T Correia, Inês C Conceição, Bárbara Oliveira, Joana Coelho, Inês Sousa, Ana F Sequeira, Joana Almeida, Cátia Café, Frederico Duque, Susana Mouga, Wendy Roberts, Kun Gao, Jennifer K Lowe, Bhooma Thiruvahindrapuram, Susan Walker, Christian R Marshall, Dalila Pinto, John I Nurnberger, Stephen W Scherer, Daniel H Geschwind, Guiomar Oliveira, Astrid M Vicente

**Affiliations:** 1 Instituto Nacional de Saúde Doutor Ricardo Jorge, Lisbon 1649-016, Portugal; 2 Center for Biodiversity, Functional & Integrative Genomics, Faculty of Sciences, University of Lisbon, Lisbon 1749-016, Portugal; 3 Instituto Gulbenkian de Ciência, Oeiras 2780-156, Portugal; 4 Unidade de Neurodesenvolvimento e Autismo, Centro de Desenvolvimento da Criança e Centro de Investigação e Formação Clínica, Hospital Pediátrico, Centro Hospitalar e Universitário de Coimbra, Coimbra 3000-602, Portugal; 5 Institute for Biomedical Imaging and Life Sciences, Faculty of Medicine, University of Coimbra, Coimbra 3000-548, Portugal; 6 Autism Research Unit, The Hospital for Sick Children and Bloorview Kids Rehab, University of Toronto, Toronto, Ontario M5G 1X8, Canada; 7 Program in Neurogenetics, Department of Neurology, Center for Autism Research and Treatment, Semel Institute, David Geffen School of Medicine at UCLA, Los Angeles, CA 90095, USA; 8 The Centre for Applied Genomics and Program in Genetics and Genome Biology, The Hospital for Sick Children, Toronto, Ontario, M5G 0A4, Canada; 9 Departments of Psychiatry, and Genetics and Genomic Sciences, Seaver Autism Center, and the Mindich Child Health & Development Institute, Mount Sinai School of Medicine, New York, NY 10029, USA; 10 Institute of Psychiatric Research, Departments of Psychiatry and Medical and Molecular Genetics, Indiana University School of Medicine, Indianapolis, IN 46202, USA; 11 Department of Molecular Genetics and the McLaughlin Centre, University of Toronto, Toronto, ON M5S 1A1, Canada

**Keywords:** *ANXA1*, Autism, Brain homeostasis, Copy number variants, Duplication, Glucocorticoids

## Abstract

**Background:**

Validating the potential pathogenicity of copy number variants (CNVs) identified in genome-wide studies of autism spectrum disorders (ASD) requires detailed assessment of case/control frequencies, inheritance patterns, clinical correlations, and functional impact. Here, we characterize a small recurrent duplication in the annexin A1 (*ANXA1*) gene, identified by the Autism Genome Project (AGP) study.

**Methods:**

From the AGP CNV genomic screen in 2,147 ASD individuals, we selected for characterization an *ANXA1* gene duplication that was absent in 4,964 population-based controls. We further screened the duplication in a follow-up sample including 1,496 patients and 410 controls, and evaluated clinical correlations and family segregation. Sequencing of exonic/downstream *ANXA1* regions was performed in 490 ASD patients for identification of additional variants.

**Results:**

The *ANXA1* duplication, overlapping the last four exons and 3’UTR region, had an overall prevalence of 11/3,643 (0.30%) in unrelated ASD patients but was not identified in 5,374 controls. Duplication carriers presented no distinctive clinical phenotype. Family analysis showed neuropsychiatric deficits and ASD traits in multiple relatives carrying the duplication, suggestive of a complex genetic inheritance. Sequencing of exonic regions and the 3’UTR identified 11 novel changes, but no obvious variants with clinical significance.

**Conclusions:**

We provide multilevel evidence for a role of *ANXA1* in ASD etiology. Given its important role as mediator of glucocorticoid function in a wide variety of brain processes, including neuroprotection, apoptosis, and control of the neuroendocrine system, the results add *ANXA1* to the growing list of rare candidate genetic etiological factors for ASD.

## Background

Family and twin studies strongly support a genetic predisposition for autism spectrum disorders (ASD), a neurodevelopmental disorder characterized by deficits in social interaction, communication, and repetitive behaviour [1,2]. However, no genes capable of explaining the majority of cases have been identified to date.

While a prevalent hypothesis has been that ASD risk results from the interaction of multiple common gene variants, each with a small effect on disorder risk [1,2], in recent years candidate gene studies, genome-wide array screenings, and exome sequencing have brought rare variants to the attention of researchers [2-6]. Rare mutations in specific genes segregating with disorders in families with ASD and/or intellectual disability (ID) have been reported, including *SHANK3, NLGN3* and *4*, *NRXN1*, and many others [7-9]. More recently, exome sequencing has uncovered variants in other genes, such as *CHD8*, *GRIN2B*, and *SCN1A*, in ASD individuals [10-12].

Studies from large research groups such as the Autism Genome Project (AGP) international consortium, have highlighted the importance of highly penetrant, rare submicroscopic deletions and duplications, designated copy number variants (CNVs), in autism etiology [13,14]. These submicroscopic CNVs, ranging from 1 kb to 10 mb, occur frequently in the human genome, and thus can contribute to genetic diversity and genomic evolution and influence disease risk [13,15]. The AGP study showed that ASD patients have a significantly higher burden of rare genic CNVs, *de novo* and inherited, when compared to control subjects. The identified CNVs frequently overlapped genes previously implicated in ASD and ID, but also implicated novel genes like *SHANK2*, *SYNGAP1*, or *DLGAP2*. Interestingly, target genes seemed to converge in a small number of affected pathways, with an enrichment of CNVs disrupting functional gene sets involved in cellular proliferation, projection, and motility as well as GTPase/Ras signalling [13].

Because CNVs frequently delete or duplicate brain expressed genes of relevance for autism, it is reasonable to assume that many are likely of pathogenic significance and altogether may explain a substantial fraction of ASD risk [16]. The rigorous assessment of the clinical consequences of CNVs, however, requires the establishment, in large population samples, of recurrence rates in patients, clinical correlations, segregation in families, comparison of frequencies with control databases, and molecular and functional studies.

To assess the clinical significance of rare CNVs identified by the AGP study, we selected, for further characterization, *de novo* or inherited CNVs that were recurrent in ASD patients but absent or extremely rare in population-based control datasets. Here, we report a small recurrent CNV duplicating a segment of the annexin A1 gene (*ANXA1*) in ASD subjects, and its detailed characterization, including frequency in patients and controls, recurrence rates, segregation in families, and breakpoint identification. We further describe the exonic and downstream region sequencing of this gene in a second ASD sample. Annexin A1, previously known as lipocortin 1, is a 37 kDa protein belonging to the annexin protein superfamily. Annexin A1 was initially identified as a potent anti-inflammatory protein, mediating glucocorticoid (GC) actions in the host defence system [17]. Its functional activities, however, far exceed this early discovery, and include cell migration, differentiation, and proliferation, regulation of cell death signalling, phagocytic clearance of apoptotic cells, and carcinogenesis. Annexin A1 has been detected in the brain, where it is thought to have a neuroprotective and anti-inflammatory function [18], and is strongly implicated in the regulation of the neuroendocrine system, in particular the hypothalamus–pituitary–adrenal (HPA) axis control by GCs [19].

## Methods

### CNV identification and characterization

#### Discovery sample

Initial screening for rare, potentially pathogenic CNVs was performed using data from the genome-wide CNV scan carried out by the AGP consortium [13]. CNV data were available for 2,147 ASD patients of European ancestry that passed all quality control filters. These subjects were recruited at centres in North America and Europe and assessed using the Autism Diagnostic Interview-Revised and Autism Diagnostic Observation Schedule, as previously described [20]. The Autism Simplex Collection database, established in a parallel project, is available for part of the study dataset and includes comprehensive clinical information with detailed diagnostic evaluation and neuropsychological profiling of patients and relatives. To ascertain the prevalence of the CNVs in control individuals, a set of 4,964 population-based controls from available databases were used for comparison [13]. This set included 1,234 controls from Ottawa (OHI) [21], 1,123 controls from northern Germany (PopGen) [22], 1,287 controls recruited by the Study of Addiction: Genetics and Environment (SAGE) consortium [23], and 1,320 controls from the Children’s Hospital of Philadelphia (CHOP) [24].

The patients and controls were genotyped using variable SNP genotyping platforms, with the characteristics and SNP distribution shown in Additional file 1. Patients and their parents were genotyped with the Illumina Infinium 1 M-single SNP or the Illumina 1 M-duo arrays [25], which include 8 and 11 probes, respectively, within the region analyzed in the present study. CNVs were analyzed using iPattern and QuantiSNP [26] detection algorithms as previously described [13]. The control genotyping data was obtained using Affymetrix Genome-Wide Human SNP 6.0 array [21,22] and Illumina Infinium 1 M-single SNP and 550 K BeadChip array [23,24] platforms (Additional file 1). Calling parameters and algorithms were the same for patients and controls, and all *ANXA1* duplications were subsequently validated using other methods. The platforms used for genotyping patients and controls have a good coverage of at least three of the four duplicated exons (exons 10, 11, and 12) and thus adequately cover the target region (Additional file 1). The Affymetrix Genome-Wide Human SNP 6.0 array, Illumina Infinium 1 M-single, and Illumina 1 M-duo SNP arrays include, respectively, 6 SNPs and 3 CNV, 8 SNPs, and 11 SNPs probes within the target region. The Illumina 550 K BeadChip array includes only 5 SNP probes, but was able to detect the duplication in several patients from a follow-up sample (see below) which was subsequently validated by qPCR, indicating that this platform, with the smallest number of probes, can adequately detect the *ANXA1* duplication.

#### Follow-up sample

A follow-up patient sample of 1,496 subjects was screened for the *ANXA1* duplication, including individuals recruited in Portugal (n = 74) [27], individuals from the Autism Genetics Resource Exchange collection (AGRE, http://www.agre.org) (n = 1,123), and non-European individuals from the AGP consortium genome-wide CNV scan [13] (n = 299). These patients were diagnosed using the same tools and protocols as the discovery sample. Extensive phenotypic information, including morphologic, cognitive, and adaptive functioning and language measures were available for these patients, as well as basic family history. Autism-related behavioural traits assessed using the Social Responsiveness Scale (SRS) [28] and the Personality Styles and Preferences Questionnaires (PSPQ) [29], were available for some relatives. A total of 410 Portuguese control individuals, not self-reporting an ASD diagnosis, were recruited from health centres and hospitals throughout the country. Informed consent was obtained from all families included in the discovery and follow-up samples, and procedures had approval from institutional review boards.

#### Ancestry analysis

Ancestry analysis was carried out using multidimensional scaling as implemented in PLINK (Purcell 2007), utilizing 90,000 autosomal SNP genotypes that were common between the Affymetrix Genome-Wide Human SNP 6.0 array and the Illumina Arrays; 1,397 unrelated HapMap3 samples (typed on the Affymetrix Genome-Wide Human SNP 6.0 array) were used as the reference set to infer ethnicities of the cases and controls (including 101 Indian, 497 African, 86 Mexican, 246 Chinese, 165 CEPH, 87 African-American, 102 Italian, and 113 Japanese). Further, 1,287 controls from the SAGE consortium, 1,234 from the Ottawa OHI and 1,123 controls from the PopGen studies were plotted with the 26 patients and relatives for whom genome-wide data was available. The CHOP control dataset was not available for ancestry analysis.

### CNV validation and screening

Putative *ANXA1* duplications identified in the discovery sample were validated by qPCR using either a pre-designed Taqman® copy number assay (Applied Biosystems, Hs01220953_cn (chr9:74973810, NCBI Build36, hg18)) or SYBR-Green I-based real time qPCR (Roche, catalogue # 04707516001). For the Taqman assay, all samples were tested in quadruplicate, and qPCR reactions were performed as duplex reactions with RNase P (Applied Biosystems VIC-TAMRA dual labelled probe) as the reference assay, according to the manufacturer’s instructions, on an Applied Biosystems 7900 HT Real Time PCR machine. Results were analyzed using Copy Caller software (v.1.0, Applied Biosystems, USA). SYBR-Green I-based qPCR was performed using two independent primer pairs designed at the *ANXA1* locus and at the *FOXP2* locus on chromosome 7 as a diploid control.

Screening of *ANXA1* duplications in the Portuguese follow-up sample or control subjects was performed using the same Taqman assay or by Long Range PCR using a SequalPrep Long PCR kit (Invitrogen). Primers were designed using Primer3 software [30]. In the remaining follow-up population, CNVs previously identified by the AGP or by AGRE (called by PennCNV [31] using the Illumina 550 K BeadChip [5] or Omni-1 Quad genotypes) (Geschwind lab, unpublished data) were confirmed using SYBR-Green I-based qPCR performed on a LightCycler 480 Real-Time PCR system. RNase P was used as a reference gene and a pooled DNA sample from 94 healthy individuals as calibrator for relative quantification.

### Breakpoint mapping

For breakpoint mapping, *ANXA1* duplications were amplified using Long Range PCR and PCR products were sequenced in both directions using fluorescent dye terminators (BigDye Terminator v1.1 Cycle Sequencing Kit, Applied Biosystems, Forest City, CA, USA) and the same PCR primers on the ABI3730xls DNA Analyzer (Applied Biosystems).

### Screening for sequence variants

#### Sample

Sequencing of the *ANXA1* coding and downstream regions was performed in a population sample of 490 ASD Portuguese patients recruited and diagnosed as described above, including all patients previously screened by the AGP for CNVs. The frequency of selected variants of particular relevance was estimated in 262 healthy blood donors untested for ASD, with no family history of neuropsychiatric diseases, recruited in Portugal.

#### Sequencing of coding region and exon/intron boundaries

The 13 exons, the corresponding exon/intron boundaries and two conserved non-coding regions (chr9:74957159–74957417; chr9:74968961–74969803, NCBI Build36, hg18) of the *ANXA1* gene were sequenced using Roche 454 massively parallel DNA sequencing. Oligonucleotide primers, tagged with sequencing adaptors and different multiplex identifiers (MIDs) of 10 nucleotide bases, were designed for amplification of 21 *ANXA1* gene fragments (average length of 390 bp) using Primer3 [30] and OligoExplorer (Gene Link) software.

Genomic DNA of the patients was accurately quantified by fluorimetry (Quanti-iT PicoGreen dsDNA Assay Kit, Invitrogen) and then grouped in nine equimolar pools. The nine pools were independently used as templates for amplification of the 21 fragments using primers tagged with different MIDs. Amplification reactions, in a total of 189, were performed with FastStart High Fidelity Taq DNA Polymerase (Roche). The amplicons were purified with High Pure 96 UF Cleanup Plates (Roche), visualized in an automated capillary electrophoresis system (Caliper Life Sciences), quantified by use of PicoGreen dsDNA quantitation reagent, and mixed in equimolar pools for clonal amplification by emulsion PCR.

Resulting DNA library beads were loaded into the wells of a PicoTiterPlate device and run in the Genome Sequencer FLX Instrument. Nucleotide reads obtained by massively parallel sequencing were aligned to the reference sequence using Amplicon Variant Analyzer software (Roche). Upon identification of predicted differences between reads and reference, the variants were further analyzed taking into account the following criteria to select high confidence variants: i) the number of reads harbouring the alteration was above the expected value for one allele (one heterozygous individual) in the forward or in the reverse reads and ii) the alteration was detected with both forward and reverse nucleotide reads.

Selected variants with increased frequency in cases vs*.* control databases were subsequently individually genotyped using Taqman Custom genotyping assays in an ABI PRISM 7900 HT sequence detector system (Applied Biosystems) or Sequenom IPLEX assays with allele detection by mass spectroscopy, using Sequenom MassARRAY technology (Sequenom, San Diego, CA, USA). For the later, primer sequences were designed using Sequenom’s MassARRAY Design 3.0 Software and are available upon request.

#### Downstream gene region sequencing

The 3’ region of *ANXA1* (chr9:74975115–74978071, NCBI Build36, hg18) was sequenced by Sanger sequencing (primers available upon request). Contigs were assembled and sequences were aligned using the GAP program v4.11.2 from Staden package [32].

#### Bioinformatic prediction of variant effect

Functional impact of novel unique ASD variants was assessed using several prediction tools. Human Splicing Finder [33] and ESE-FINDER [34] were used to investigate potential effects on splicing. Putative changes in transcription factor and microRNA binding sites were assessed using TRANSFAC [35] and miRANDA [36], respectively. Conservation of orthologous positions across diverse species was investigated using Phastcons [37] and overlap with experimental regulatory features was examined on the UCSC Genome browser [38].

## Results

**Figure 1:**
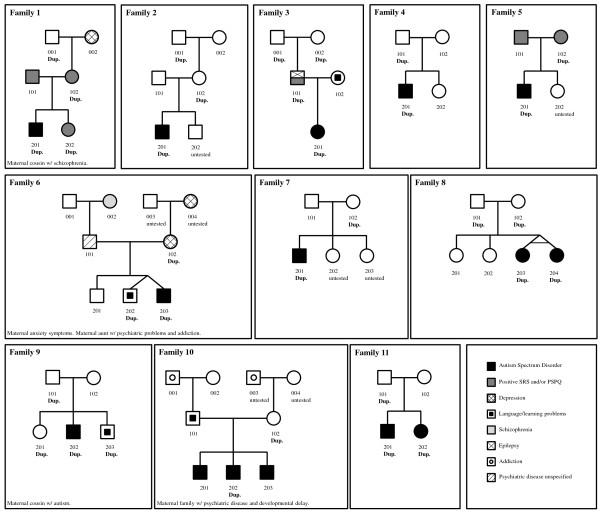
**Pedigrees of the 11 unrelated autistic patients and their affected (n = 2) and unaffected relatives carrying the *ANXA1 *duplication.** Families 1–5 were part of the AGP whole genome study, while families 6–11 were identified in the follow-up study. All available relatives were tested for the *ANXA1* duplication. Dup., individuals carrying the duplication. Untested, individuals for which no DNA sample was available.

The *ANXA1* gene includes 13 exons, encoding four protein-coding transcripts (ENST00000257497, ENST00000376911, ENST00000415424 and ENST00000456643), the largest of which is transcribed from all 13 exons. A duplication encompassing the last four exons of the *ANXA1* gene was identified in 5 out of 2,147 unrelated patients from the AGP whole genome study (Families 1–5 in Figure 1). In 4,964 population-based controls from available databases, we did not find this duplication (*P* = 0.0025). Ancestry analysis (Additional file 2) clustered together the 5 AGP patients presenting the duplication with 3,558 European samples from the SAGE, PopGen, and OHI control populations. Restricting the analysis to these ancestry-matched cases and controls, we still found a significant difference in the frequency of the duplication between cases and controls (*P* = 0.0075).

In a follow-up sample including 1,496 ASD patients and 410 control subjects, the *ANXA1* duplication was detected in 6 unrelated affected individuals (from Families 6–11 in Figure 1) and none of the controls. Ancestry analysis of these subjects showed that most patients and relatives from the AGRE dataset were spread with non-European SAGE and OHI controls, with a few overlapping with Mexican populations, as expected since some of these patients are Caucasians of Hispanic ethnicity (Additional file 2).

The overall prevalence of the *ANXA1* duplications was estimated at 11/3,643 (~0.30%) in unrelated ASD patients, in contrast with 0/5,374 in controls (*P* = 4.64 × 10^5^). Both the patient, relative, and control samples had a heterogeneous ancestry, and therefore we did not find evidence for a population effect that could explain the discrepancy of frequency of *ANXA1* duplication in patients and controls.

### No distinctive clinical phenotype in *ANXA1* duplication carriers

The 13 patients in 11 families identified with the duplication met criteria for autism or ASD diagnosis (Table 1), although the clinical phenotype of the patients carrying the duplication was heterogeneous. Intellectual level ranged from normal in 2 patients to moderate ID in 5 subjects (in 4 patients IQ level was not known). Regarding language function, 9 patients were verbal, but 5 of these presented phrase speech delay, while the remaining 4 presented with severe language impairment, most using only isolated words. Other language abnormalities, such as articulation problems, abnormal prosody and modulation, stuttering, hyperlexia or apraxia were less common. Neurological dysfunction such as seizures, hypotonia or dyskinesias, as well as minor dysmorphologies and language or developmental regression, was present in 5 of the patients. Three patients showed other associated problems, such as mitochondrial dysfunction and gastrointestinal or sleep problems.

**Table 1:** **Clinical phenotype of the ASD patients with the identified *ANXA1 *** **duplication**

**ID**	**Sex**	**Geographical origin**	**Reported ancestry**	**ASD type**	**IQ level**	**Language**	**Motor, neurological, and sensory problems**	**Physical exam**	**Developmental history**	**Relevant medical history**	**Family type**	**Duplication inheritance**
Fam1_201	Male	Portugal	European	Autism	Moderate ID	Phrase speech delay; hyperlexia	No	Normal	No regression; psychomotor development delayed	Possible mitochondrial disease; sleep problems; rumination	SPX	Maternal
Fam2_201	Male	Portugal	European	Autism	Mild ID	Abnormal speech; only isolated words	No	Normal	No regression; psychomotor development delayed with an onset at 2 years	None	UNK	Maternal
Fam3_201	Female	Portugal	European	ASD	Normal IQ	No speech delay	Clumsy child	Myopia	No regression and no psychomotor delayed development	NA	SPX	Paternal
Fam4_201	Male	Canada	European	Autism	Moderate ID	Severe language impairment; speech and oral motor deficit (i.e., apraxia); uses single words	Possible history of seizures	Normal	No regression	NA	UNK	Paternal
Fam5_201	Male	USA	European	Autism	Mild ID	Phrase speech delay	No	Normal	No regression	None	SPX	Maternal
Fam6_203	Male	USA	European	Autism	Moderately impaired or delayed	Verbal; no speech delay; articulation problems; abnormal prosody and modulation; stuttering; extreme to moderate low score on PPVT	Gait abnormalities; repetitive movements (finger; knocking); sensory abnormalities; abnormal light touch; tactile defensiveness; dyskinesias elicited lateral foot walking	Epicanthal folds; left absent tragus; *café au lait*	Language regression at 15 months	Gastroesophageal reflux; chronic diarrhoea and constipation; allergies and food sensitivity; sleep problems	SPX	Maternal
Fam7_201	Male	Portugal	European	Autism	Normal IQ	No speech delay	No	Normal	No regression; psychomotor development delayed	Sleep problems	UNK	Maternal
Fam8_203	Female	USA	Hispanic/Latino	Autism	NA	Verbal; phrase speech delay	NA	NA	No regression	NA	SPX	Both
Fam8_204	Female	USA	Hispanic/Latino	Autism	NA	Verbal; phrase speech delay	NA	NA	No regression	NA	SPX	Both
Fam9_202	Male	USA	Hispanic/Latino	Autism	NA	Verbal; phrase speech delay	NA	NA	No regression	NA	MPX	Paternal
Fam10_202	Male	USA	Hispanic/Latino	Autism	NA	Non-verbal	Gait abnormalities; repetitive movements (hand flapping, finger movements, body rocking); increased acoustic and tactile sensibility; tactile defensiveness	Slanted posterior fontanel; low set and posterior angulation ears; bifid uvula; high arched palate; finger clinodactyly	No regression	Neonatal hyperbilirubinemia and anaemia	MPX	Maternal
Fam11_201	Male	USA	Hispanic/Latino	Autism	Moderately impaired or delayed	Verbal; no speech delay; extreme to moderate low score on PPVT	NA	Normal	Developmental and language regression	NA	MPX	Paternal
Fam11_202	Female	USA	Hispanic/Latino	Autism	Moderately impaired or delayed	Non-verbal; phrase speech delay; extreme to moderate low score on PPVT	NA	Normal	Regression	NA	MPX	Paternal

PPVT, Peabody Picture Vocabulary Test; NA, no information available; SPX, simplex; MPX, multiplex; UNK, unknown.

To search for potential multiple hits that might modulate clinical expression, genome-wide CNVs were analyzed in the 5 individuals carrying the *ANXA1* variant for which this data was available. No additional CNVs with an overlap of less than 50% with controls were identified that were common between these 5 individuals.

### All duplications are inherited

Family analysis showed that the duplication was inherited in all 13 carrier affected individuals (Figure 1). In 6 patients, the CNV was inherited from the mother, in 5 from the father, and in the remaining 2 patients, who are monozygotic twins, both parents carried the duplication. No consanguinity has been reported in this family, and the twins had one copy of the duplication each. Grandparents were available for three families, and maternal grandfather transmission was observed in two families (1 and 2), whereas in the third family (family 3) both paternal grandparents carried the duplication (Figure 1). These grandparents were reportedly distant cousins, so there may have been a degree of consanguinity in the family.

Ascertainment of family history further established that all 13 patients had a positive family history of intellectual or neuropsychiatric problems, with cases of ASD, language and learning disability, schizophrenia, depression, and addiction among first or second-degree relatives (Figure 1). In the three families (1, 3, and 5) where autism traits in parents were evaluated using the SRS [28] and PSPQ [29] questionnaires, the transmitting parent scored positive for at least one of these scales. Two affected (family 10) and 8 unaffected siblings (families 1, 4, 6, 8, and 9) were also available for testing. Two of the 3 affected siblings from family 10 did not carry the duplication. However, this family is heavily loaded in psychopathology both on the paternal and maternal sides and it is thus conceivable that multiple autism-associated variants are segregating in this sibship. Four out of the 8 unaffected siblings also carried the duplication (families 1, 6, and 9). Nevertheless, a closer inspection of the clinical phenotype showed that 3 of the 4 unaffected siblings carrying the duplication had social interaction or cognitive problems: a positive SRS of clinical significance (proband’s sister in family 1), documented spelling difficulties and abnormal social behaviour causing parental concern (dizygotic twin in family 6), and language/speech and learning disabilities requiring therapy and educational support (proband’s brother in family 9). Only the proband’s sister in family 9 carries the duplication but has no indication of any behavioural problem, suggesting that incomplete penetrance and/or modulation by other factors may occur. The remaining 4 tested siblings (in families 4, 6, 8) not presenting the *ANXA1* duplication did not have any psychopathologic diagnosis or cognitive disability. The variability in autism traits, psychopathology, and cognitive deficits in siblings and parents is concordant with the heterogeneity of symptoms in the affected duplication carriers, and more broadly, the notion of complex genetic inheritance [39].

### Same *ANXA1* breakpoints in all carriers

**Figure 2:**
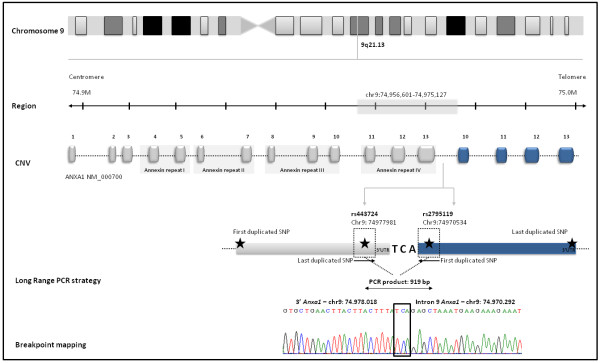
**Genomic characterization of *ANXA1 *duplication: gene location and structure.** We performed a Long Range PCR to determine whether the duplication was in *tandem*, using primers pointing outwards from the location of the first and last duplicated SNP. The four duplicated exons are represented in blue. A sequence of microhomology of three nucleotides (TCA) was also identified and is probably mediating the duplication.

A PCR assay with primers pointing outwards from the location of the first and last duplicated SNP in the Illumina Infinium 1 M-single SNP array confirmed that the duplication was in *tandem* and in the direct orientation (Figure 2). Sequencing of this PCR product defined the breakpoints of the duplication and determined its size (7,728 base pairs, spanning chr9:74970292–74978018 from NCBI Build 36, hg18; Figure 2). Although the predictions by PennCNV and QuantiSNP were not the same for all individuals [13], breakpoints were found to be identical in the 13 ASD probands and 15 relative carriers, suggesting a single ancestral event. The distal breakpoint resides in intron 9 (chr9: 74970292; Figure 2), while the proximal end is located 2,891 bp downstream of the gene (chr9: 74978018; Figure 2). A sequence of microhomology of 3 nucleotides (TCA) was present in all the individuals at both breakpoints, and is probably mediating the duplication (Figure 2). The haplotype analysis of a window of 44 SNPs common between the various genotyping platforms, downstream (about 111 kb) and upstream (about 133 kb) of the duplication, in the 10 probands with genome wide-data available, was done by comparing the haplotypes of the probands, two by two, and calculating the similarity for each pair. The haplotypes flanking the duplication have an average 88% similarity.

### Identification of novel *ANXA1* sequence variants

#### Exonic and splice site regions

To screen for additional variants in the *ANXA1* gene that could be conferring risk for autism, sequencing of the 13 exons, adjacent intron boundaries and the 3’region downstream of the gene was carried out in a cohort of 490 Portuguese ASD patients.

Exon and exon/intron boundaries were sequenced using a DNA pooling approach. A total of 735,987 nucleotide reads were obtained, corresponding to a mean coverage of 73× per fragment per individual. Forty-two alterations were identified by sequencing of exonic and splice site regions, 32 of which were considered high confidence (see Methods for criteria) and were further considered.

Based on an increased frequency of the variant in cases vs. control databases, 28 of the 32 variants were selected for validation by individual genotyping in the same ASD sample and in 262 Portuguese control individuals. Five rare variants that were not listed in SNP databases and were absent in the panel of 262 control individuals were identified (Table 2). Four of these variants were intronic and *in silico* analysis did not determine any putative functional role; one variant was located in the region upstream of the gene. This latter variant mapped to a conserved residue 48 bp upstream of the transcription start site and overlapped with several experimentally confirmed regulatory features, such as a DNA hypersensitivity cluster, transcription binding sites, and histone marks, suggesting a potential regulatory role. TRANSFAC [35] analysis predicted potential alterations in transcription factor binding sites, including the abolishment of a binding site for the vitamin D receptor and the creation of an orthodenticle homeobox binding site (OTX), previously implicated in psychiatric disorders [40,41]. Additionally, 3 SNPs absent in the control sample and described as monomorphic in the Hapmap CEU population from dbSNP were further evaluated for a potential functional role (Table 2). One SNP (rs2795115) was located in the 5’UTR region but no functional alterations were predicted by *in silico* analysis. Another SNP (rs10119605) was located in intron 11–12, and a third one (rs10114350) was mapped to a splice site sequence 7 bp upstream of exon 4, in a highly conserved position in primates and altering an intronic splicing enhancer.

**Table 2:** **Identified *ANXA1 *** **novel variants and SNPs absent in controls**

**Genomic position (hg18)**	**Nucleotide change**	**Amino acid change**	**Location**	**Transcript**	**Novel/SNP**	**Frequency cases**	**Genotype**	***In silico *** **analysis**	**PhastCons (primates)**
74956445	G > A	–	5' of the gene	ENST00000257497	Novel^1^	0.002	Homo	TFBS abolished: VDR, CAR, PXR; NF-Y, C-myb, Crx; TFBS created: HNF6, Nkx6-2, OTX, CdxA	0.48
74956718	CTA > Del.CTA	–	Intron 1–2	ENST00000257497	Novel^1^	0.002	Het	-	0.23
74962617	C > T	–	5'UTR	ENST00000376911	rs2795115^1^	0.002	Het	-	0.014
74964058	T > C	–	intron 3–4	ENST00000257497	rs10114350^1^	0.002	Het	splice-site SNP (ENSEMBL), ISRE down (pfSNP)	0.81
74972178	G > A	–	intron 10–11	ENST00000257497	Novel^1^	0.005	Het	–	0.1
74972626	T > G	–	Intron 11–12	ENST00000257497	Novel^1^	0.002	Het	–	0.18
74973075	G > C	–	Intron 11–12	ENST00000257497	rs10119605^1^	0.002	Het	–	0.11
74973545	T > C	–	Intron 11–12	ENST00000257497	Novel^1^	0.002	Het	–	NA
74975361	G > A	–	3' (intergenic)	–	Novel	0.002	Het	–	0.05
74975609	T > A	–	3' (intergenic)	–	Novel	0.002	Het	–	0.78
74975645	T > C	–	3' (intergenic)	–	rs149272288	0.001	Het	–	0.014
74975873	C > T	–	3' (intergenic)	–	rs9792653	0.051	Het	–	NA
74975882	C > T	–	3' (intergenic)	–	rs143327464	0.004	Het	–	NA
74976171	C > T	–	3' (intergenic)	–	rs113627562	0.004	Het	–	NA
74976222	A > G	–	3' (intergenic)	–	rs72737044	0.017	Het/Homo	–	0.18
74976536	C > T	–	3' (intergenic)	–	Novel	0.001	Het	–	0.03
74976574	T > C	–	3' (intergenic)	–	rs78798837	0.002	Het	–	NA
74977215	T > C	–	3' (intergenic)	–	Novel	0.001	Het	–	0.006
74977218	C > T	–	3' (intergenic)	–	rs146061737	0.001	Het	–	0.003
74977655	A > G	–	3' (intergenic)	–	rs114240435	0.002	Het	–	0
74977680	C > A	–	3' (intergenic)	–	rs114833327	0.001	Het	–	NA
74977840	G > A	–	3' (intergenic)	–	rs11143512	0.465	Het/Homo	–	NA
74977852	A > G	–	3' (intergenic)	–	Novel	0.001	Het	–	0.01
74977888	C > G	–	3' (intergenic)	–	rs17653109	0.089	Het/Homo	–	0.07
74977928	C > T	–	3' (intergenic)	–	rs75260654	0.023	Het	–	NA
74977965	C > G	–	3' (intergenic)	–	Novel	0.001	Het	–	0.08
74977981	C > T	–	3' (intergenic)	–	rs4443724	1	Homo	–	0.003
74977990	C > T	–	3' (intergenic)	–	rs116224215	0.007	Het	–	NA
74978003	C > T	–	3' (intergenic)	–	rs4285546	0.04	Het/Homo	–	0.003

^1^Absent in a panel of 262 control individuals; Homo, homozygous; Het, heterozygous; TFBS, transcription factor binding site; NA, not available.

#### Intergenic region

The 3’ region downstream the 3’UTR of *ANXA1*, which is also included in the duplication and spans approximately 3,000 bp, has 31 nucleotide positions varying between *H. sapiens* and *P. troglodytes*. Considering the mean autosomal single nucleotide divergence between these two species (~1.33%; [42]), which would predict 39 nucleotide changes, we observed a trend toward a purifying selection in this region. Sequencing identified a total of 21 variants, 15 of which were previously reported SNPs and 6 were novel variants found in 8 individuals (Table 2). One of these novel variants was exclusive of 2 monozygotic twins (both affected). One of the 6 novel variants was highly conserved, however none of the 6 changes had predicted functional consequences by *in silico* analysis using SNP Nexus [43] and TRANSFAC [35], suggesting that these are probably neutral variants.

## Discussion

In this study, a 7.7 kb inherited duplication on chromosome 9q21.13, encompassing the four last exons of the *ANXA1* gene, was identified in 11 out of 3,643 unrelated autistic patients but in none of 5,374 healthy controls. Ancestry analysis did not provide evidence for a population stratification effect explaining the presence of the duplication exclusively in patients. We also carefully assessed the possibility that absence in control samples from available databases was the result of false negatives due to genotyping platform differences. SNP coverage was adequate in the duplicated region in several of the platforms (8 to 11 SNPs), while the array targeting the smallest number of SNPs was able to detect the duplication in some of the patients. The intriguing duplication frequency in cases versus its absence in controls thus prompted us to further investigate the potential contribution of variants in this gene for autism etiology.

As is commonly observed in ASD, the identified *ANXA1* duplication was not associated with a distinctive phenotype, but patients showed a heterogeneous clinical presentation in terms of ASD phenotype, intellectual disability, language difficulties, neurodevelopmental regression, or dysmorphic features [44-46]. A clear pattern of transmission of the duplication with ASD was not observed; however, a more meticulous analysis of the phenotype in available relatives showed that many parents and siblings carrying the duplication present a broader autism phenotype, language disability, cognitive deficits, or neuropsychiatric problems. One single family included cases of ASD with and without the duplication. However, this family was heavily burdened with neuropsychiatric disease on both the maternal and paternal side, and therefore it is plausible that several etiological factors affected the family simultaneously. In light of present day literature, these observations suggest that a low penetrance *ANXA1* duplication may be associated with a broader autism phenotype and co-morbidities, with other unidentified factors interacting with the duplication to influence its phenotypic expression. Supporting this possibility, there is growing evidence indicating that, in addition to clinical overlap between clinical entities in the neuropsychiatric spectrum and ASD [28,47], shared heritability and overlapping genetic factors may lead to variable expressivity and incomplete penetrance in families of ASD subjects [48-52].

Taking into consideration the multiple hit model proposed for ASD [53,54], we searched for modulating risk or protective genetic factors that might regulate the clinical expression of the *ANXA1* duplication. No other CNV in common between these individuals was identified in the AGP whole genome study suggesting that a double hit is a rare occurrence or that such modulating factors are heterogeneous among these patients. Future exome sequencing will help clarify this issue.

The exact same location of the breakpoints in all duplication carriers indicates that it is likely an ancestral event – a hypothesis that is further supported by the similar haplotypes flanking the duplication. The region of microhomology is consistent with studies showing that the breakpoints in 40% of duplications and 70% of deletions had regions with 1 to 30 bp of microhomology [55,56].

Extensive sequencing of exonic and regulatory regions was carried out to identify additional sequence variants in the *ANXA1* gene that might contribute to ASD etiology, as well as any changes co-occurring with the inherited CNVs. Sequencing uncovered a number of novel variants and previously reported monomorphic SNPs absent from a panel of control individuals. One variant upstream of the gene disrupts or creates binding sites for transcription factors such as the vitamin D receptor and OTX, which have been implicated in psychiatric disorders including autism [40,41], thus potentially modulating *ANXA1* expression. Based on conservation and functional *in silico* prediction tools, some of these variants are of potential interest, although no obvious pathogenic variants have been identified.

Abnormal post-translational processing of *ANXA1* has been previously observed in individuals with Fragile X syndrome [57], which frequently presents with autistic symptomatology. Expression studies will be necessary to assess if this variant alters *ANXA1* expression and to elucidate the potential impact of the duplication. The latest ENCODE results [58] show DNase I hypersensitivity clusters in the distal breakpoint region, suggesting a possible disruption of a regulatory sequence. These, and other mechanisms by which the *ANXA1* duplication can be deleterious, need to be clarified.

Annexin A1, a member of the annexin superfamily that contains 13 calcium or calcium and phospholipid-binding proteins, has been implicated in many diverse cellular functions, including anti-inflammatory effects [18,59,60], cell growth [61], apoptosis [62], membrane fusion, endocytosis, and exocytosis [63]. The consequences of annexin A1 dysregulation could therefore influence multiple pathways, some of which have been previously linked to autism pathophysiology. Annexin A1 was first identified as a GC-inducible protein and a potential mediator of the anti-inflammatory actions of these steroid hormones [17,64], ensuring an appropriate level of activation of innate immune cells [59] and/or transducing a stimulatory signal to promote T-cell activation. Immunological dysfunction has been a recognized feature in ASD, supported by the observation of abnormal levels of circulating brain autoantibodies and anti-inflammatory markers as well as neuroglial activation and neuroinflammation in several brain regions in ASD patients [65-68]. Annexin A1 also controls the non-inflammatory phagocytosis of apoptotic neurons and promotes the resolution of inflammatory microglial activation [18], thus regulating neuronal apoptosis during neurological development and the mature brain. Furthermore, annexin A1 plays a fundamental role in the regulation of the HPA axis, effecting the negative feedback of GC at the level of the pituitary gland and hypothalamus [69], and thus modulating the secretion of corticotrophin (adrenocorticotropic hormone) and its hypothalamic releasing hormones, corticotrophin-releasing hormone and arginine vasopressin [19]. There is evidence that the HPA axis, as part of the limbic system which is the neural basis for emotion and social functioning, is impaired in autistic children [70-75]. For instance, abnormal responses of autistic subject to stress as well as increased levels of cortisol secretion and adrenocorticotropic hormone in serum of autistic males have been reported [76-80]. Abnormalities in corticotropic cell number and structure in male *ANXA1* knockout mice further support this hypothesis [81].

## Conclusions

The identification of a recurrent *tandem* duplication of the *ANXA1* gene in autistic patients which is not present in a very large set of controls, supported by family observations of co-occurrence of the variant with neuropsychiatric disability, suggests an involvement of this gene in the etiology of ASD. The variety of physiological mechanisms where annexin A1 has been implicated implies a fundamental role of this molecule in brain homeostasis, with specific aspects clearly relevant for the pathophysiology of ASD. Overall, the results described herein constitute supporting evidence for *ANXA1* as one more etiological risk factor for ASD, warranting further functional investigation.

## Abbreviations

AGP: Autism Genome Project; AGRE: Autism Genetics Resource Exchange; ANXA1: Annexin A1; ASD: Autism spectrum disorders; CHOP: Children’s hospital of Philadelphia; CNVs: Copy number variants; ENCODE: Encyclopedia of DNA elements; GC: Glucocorticoid; HPA axis: Hypothalamus–pituitary–adrenal axis; ID: Intellectual disability; kb/mb: kilobase/megabase; kDa: Kilodalton; MIDs: Multiplex identifiers; OHI: Ottawa heart institute; PSPQ: Personality styles and preferences questionnaire; SAGE: Study of addiction, genetic and environment; SRS: Social responsiveness scale.

## Competing interests

The authors declare that they have no competing interests.

## Authors’ contributions

CTC and ICC carried out CNV validation, breakpoint mapping, segregation, and sequence analysis. BO, JC, IS, and AFS participated in CNV and sequence analysis and manuscript revision. KG, JKL, BT, and SW participated in CNV and phenotypic data analysis, and manuscript revision. JA, CC, FD, SM, GO, and WR recruited and performed the clinical assessment of patients’ and relatives, and revised the manuscript. CM, DP, JIN, SWS, and DHG contributed patient samples, participated in the interpretation of results and manuscript revision. AMV, CTC, and ICC carried out the study design, interpretation of data, and the drafting of the manuscript. All authors read and approved the final manuscript.

## Supplementary Material

Additional file 1 **Genotyping platform coverage of *ANXA1 *** **duplicated region.** SNPs that are common between the platforms used in the AGP discovery sample, the AGRE follow-up sample, and control datasets are represented (black triangle). SNPs covered exclusively by the Illumina 1 M-duo array (purple triangle), the Illumina Omni-1 Quad array (green triangle) and the Affymetrix Genome-Wide Human SNP 6.0 array (blue triangle) are also represented, as well as the 26 bp CNV probes (blue circle) of Affymetrix Genome-Wide Human SNP 6.0 array. Click here for file

Additional file 2 **Multidimensional scaling analysis results, using 1,397 unrelated HapMap3 samples as reference set to infer ethnicities, control samples from SAGE consortium, Ottawa (OHI), Northern Germany (PopGen), and the ASD cases and relatives with the *ANXA1 *** **duplication (AGP and AGRE).** Click here for file
