# Supplementary material for: Recurrent duplications of the annexin A1 gene (ANXA1) in autism spectrum disorders
Source: Mol Autism. 2014 Apr 10;5:28. doi: 10.1186/2040-2392-5-28 (PMC4098665; doi:10.1186/2040-2392-5-28)
Supplement: Additional file 1 — Genotyping platform coverage of ANXA1 duplicated region. SNPs that are common between the platforms used in the AGP discovery sample, the AGRE follow-up sample, and control datasets are represented (black triangle). SNPs covered exclusively by the Illumina 1 M-duo array (purple triangle), the Illumina Omni-1 Quad array (green triangle) and the Affymetrix Genome-Wide Human SNP 6.0 array (blue triangle) are also represented, as well as the 26 bp CNV probes (blue circle) of Affymetrix Genome-Wide Human SNP 6.0 array. [file 2040-2392-5-28-S1.PDF]

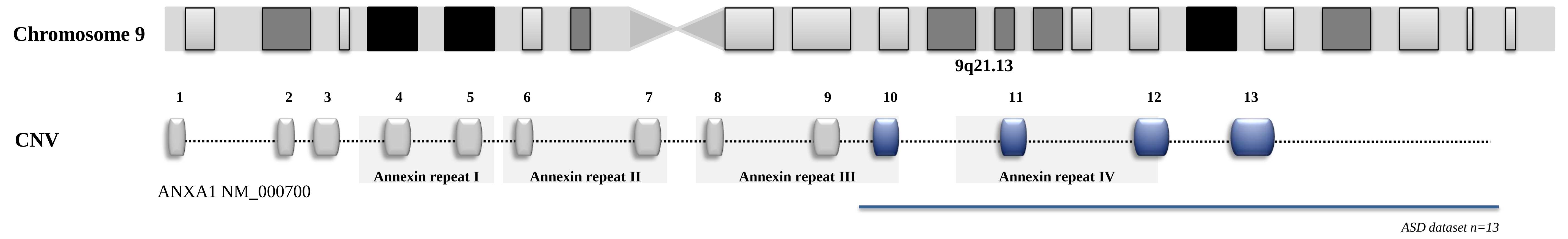

Sample	N	Genotyping platform	Reference in the main text
AGP (discovery and follow-up sample)	2446	Illumina Infinium 1M-single SNP or Illumina 1M-duo arrays	13;21
AGRE (follow-up sample)	1123	Illumina 550K BeadChip and Illumina Omni-1 Quad arrays	-
Ottawa (OHI)	1234	Affymetrix Genome-Wide Human SNP 6.0 array	23
Northern Germany (PopGen)	1123	Affymetrix Genome-Wide Human SNP 6.0 array	24
SAGE consortium	1287	Illumina Infinium 1M-single SNP array	25
Children's Hospital of Philadelphia (CHOP)	1320	Illumina 550K BeadChip array	26

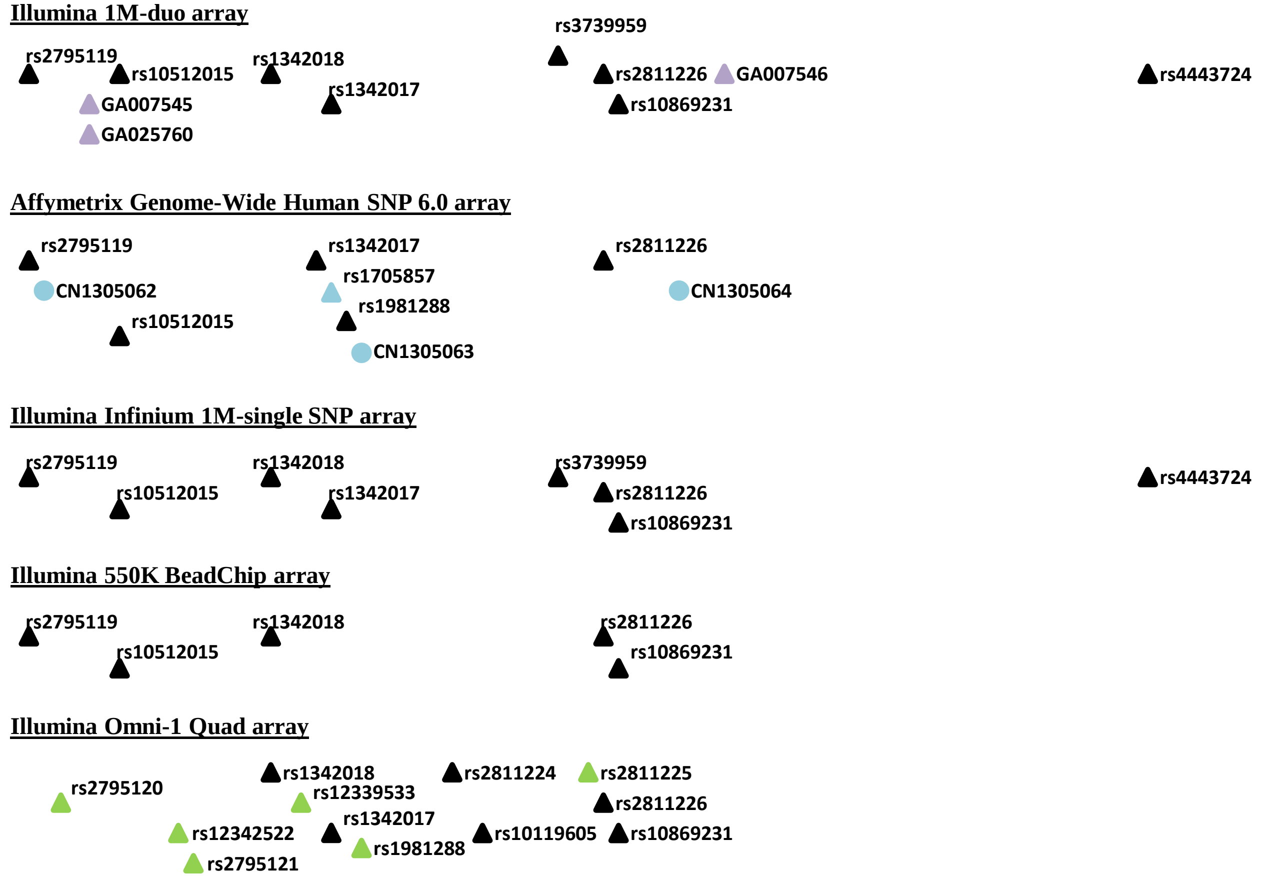

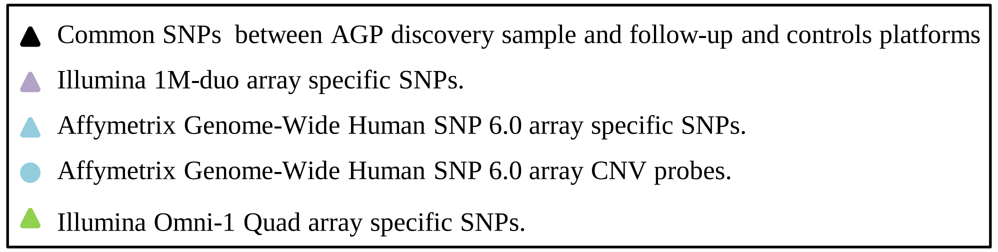

Additional File 1. Genotyping platform coverage of *ANXA1* duplicated region. SNPs that are common between the platforms used in the AGP discovery sample, the AGRE follow-up sample and control datasets are represented (black triangle). SNPs covered exclusively by the Illumina 1M-duo array (purple triangle), the Illumina Omni-1 Quad array (green triangle) and the Affymetrix Genome-Wide Human SNP 6.0 array (blue triangle) are also represented, as well as the 26 bp CNV probes (blue circle) of Affymetrix Genome-Wide Human SNP 6.0 array.

Probes genomic coordinates: rs2795119(chr9:74970534); GA007545(chr9:74970929); GA025760(chr9:74970930); rs10512015(chr9:74971066); rs1342018(chr9:74971951); rs1342017(chr9:74972330); rs3739959(chr9:74973757); rs2811226(chr9:74974084); rs10869231(chr9:74974147); GA007546(chr9:74974834); rs4443724(chr9:74977981); CN1305062(chr9:74970552-74970577); rs17058570(chr9:74972375-74972375); rs1981288(chr9:74972475); CN1305063(chr9:74972520-74972545); CN1305064(chr9:74974461-74974486); rs2795120(chr9:74971196); rs12342522(chr9:74971565); rs2795121(chr9:74971584); rs12339533(chr9:74971988); rs10119605(chr9:74973075); rs2811225(chr9:74974026); rs2811226(chr9:74974084).
