# Supplementary material for: Recurrent duplications of the annexin A1 gene (ANXA1) in autism spectrum disorders
Source: Mol Autism. 2014 Apr 10;5:28. doi: 10.1186/2040-2392-5-28 (PMC4098665; doi:10.1186/2040-2392-5-28)
Supplement: Additional file 2 — Multidimensional scaling analysis results, using 1,397 unrelated HapMap3 samples as reference set to infer ethnicities, control samples from SAGE consortium, Ottawa (OHI), Northern Germany (PopGen), and the ASD cases and relatives with the ANXA1 duplication (AGP and AGRE). [file 2040-2392-5-28-S2.PDF]

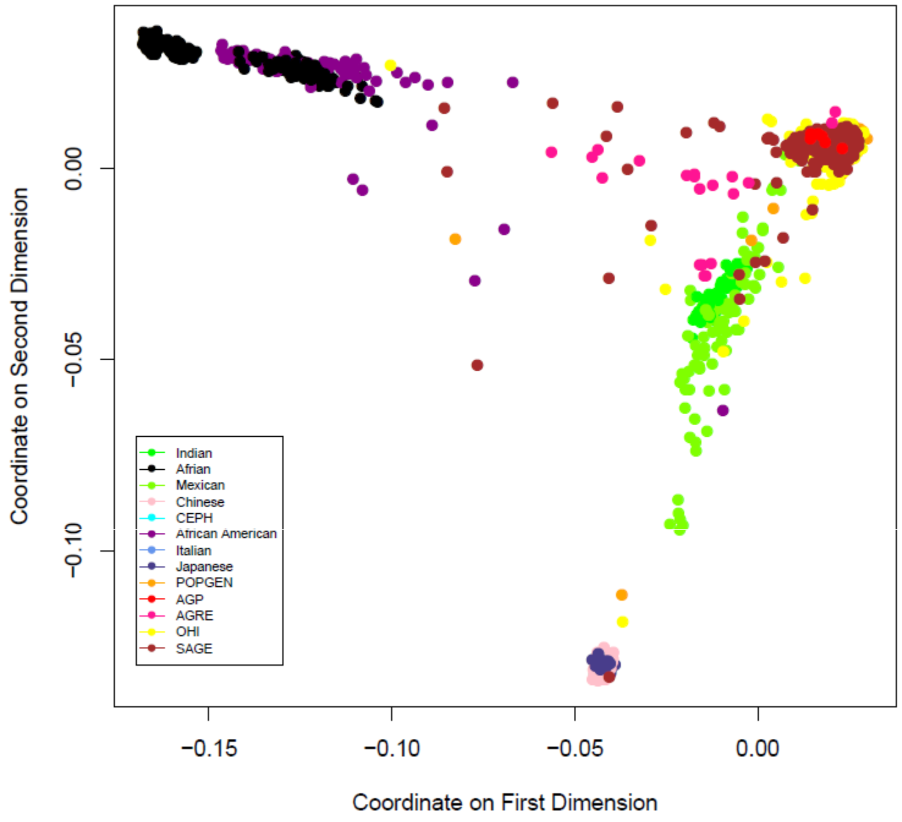

Additional File 2. Multidimensional Scaling (MDS) analysis results, using 1397 unrelated HapMap3 samples as reference set to infer ethnicities, control samples from SAGE consortium, Ottawa (OHI) and Northern Germany (PopGen), and the ASD cases and relatives with the *ANXA1* duplication (AGP and AGRE).
